# Complete chloroplast genome sequence and phylogenetic analysis of a wild species of abiu fruit, *Pouteria caimito* (Ruiz & Pavon.) Radlk

**DOI:** 10.1080/23802359.2020.1852897

**Published:** 2021-01-12

**Authors:** Liang Tao, Zhuo-gong Shi, Qing-yi Long

**Affiliations:** a Key Laboratory for Forest Resources Conservation and Utilization in the Southwest Mountains of China, Ministry of Education, Southwest Forestry University, Kunming, China; b Yunnan Institute of Tropical Crops, Xishuangbanna, China

**Keywords:** Wild species, Abiu fruit, chloroplast genome

## Abstract

Abiu fruit (*Pouteria caimito* [Ruiz & Pavon.] Radlk.) is endemic to the Amazonian region of South America, the fruit is also called yellow star apple, blueberry pie fruit and cauje. In this study, the chloroplast genome sequence of *P. caimito* was assembled and characterized using Illumina sequencing. The whole chloroplast genome of the wild species of abiu fruit is 158,916 bp, composed of four regions: a large single-copy region (88,096 bp) and a small single copy (18,620 bp) region, separated by two inverted repeat regions (26,100 bp), and the GC content is 36.83%. A total of 133 genes were annotated, including 88 that encoded proteins, eight that encoded rRNA genes and 37 that encoded tRNA genes. A maximum likelihood tree was constructed based on the sequences of chloroplast genome, the results showed that the wild species of *P. caimito* is the most closely related to *Pouteria campechiana*. This study provided abundant genomics data for the research and development of *P. caimito*.

Abiu fruit (*Pouteria caimito* [Ruiz & Pavon.] Radlk.) is endemic to the Amazonian region of South America (Sotto et al. 2002; Eduardo et al. 2007). It is widely grown in the lower eastern part of Andes from southwestern Venezuela, Para, Guyana and Brazil to Columbia, Peru and Ecuador. The fruit is also called yellow star apple, blueberry pie fruit and cauje. Abiu fruit are rich in tryptophan, threonine, lysine, Vitamin C, Vitamin B3 and other nutrients (Maia et al. 2003) and are eaten raw or used to prepare desserts (Franca et al. 2016). With the increasing impact of human activities on the environment, the wild germplasm resources of abiu fruit have sharply decreased, and these germplasm resources are imperiled. Simultaneously, the resistance of wild germplasm of abiu fruit to stress is obviously stronger than that of cultivated germplasm, and it has greater breeding value. Therefore, it is necessary to study the genetic background of wild germplasm of abiu fruit at the genomics level.

Tender leaves of the wild species of abiu fruit were collected from the Tropical Fruit Garden of Yunnan Institute of Tropical Crops (22.01681945°N 100.78920353°E) that contained abiu fruit trees that had been introduced from their native environment in Brazil. The genomic DNA was extracted using a DNeasy Plant Mini Kit (Qiagen, Venlo, The Netherlands). The purified DNA samples were stored in the ultra-low temperature specimen library at Southwest Forestry University (specimen accession number: SFU-TF-2020-0189). Genomic DNA was sequenced using an Illumina Hi-Seq 2500 Platform (Illumina, San Diego, CA, USA) to assemble the chloroplast genome. Low-quality reads were removed by FastQC (Andrews 2015), the chloroplast genome was assembled by NOVOPlasty (v.2.7.2) (Dierckxsens et al. 2016) and annotated by Geneious 8.1.7 (Kearse et al. 2012) and corrected by DOGMA (Wyman et al. 2004). The results of chloroplast genome assembly and annotation were uploaded to GenBank (http://www.ncbi.nlm.nih.gov/) with the accession number MT712131.

The whole chloroplast genome of the wild species of abiu fruit is 1,58,916 bp. Like those of other plants (Chen et al. 2020; Yu et al. 2020), the genome is also composed of four regions: a large single-copy region (LSC, 88,096 bp) and a small single copy (SSC, 18,620 bp) region, separated by two inverted repeat regions (IRs, 26,100 bp). The whole chloroplast genome is composed of 31.24% A, 31.94% T, 18.04% G, and 18.79% C, and the GC content is 36.83%. A total of 133 genes were annotated, including 88 that encoded proteins, eight that encoded rRNA genes and 37 that encoded tRNA genes.

To study the phylogenetic relationship of the wild species of abiu fruit, the complete chloroplast genome sequences of 19 plant species were aligned by MAFFT (Katoh and Standley 2013) and a maximum likelihood tree was constructed based on the complete chloroplast genome sequences by RAxML 8.0.2 (Stamatakis 2014) with 1000 bootstraps under the GTRGAMMAI substitution model. *Sambucus nigra* which belongs to the Dipsacales order was used as the outgroup. The ML phylogenetic tree (Figure 1) showed that the wild species of abiu fruit is closely related to *Pouteria campechiana* (NC_033501). This study provided abundant genomics data for the research and development of *Pouteria caimito* (Ruiz & Pavon.) Radlk.

**Figure 1.**
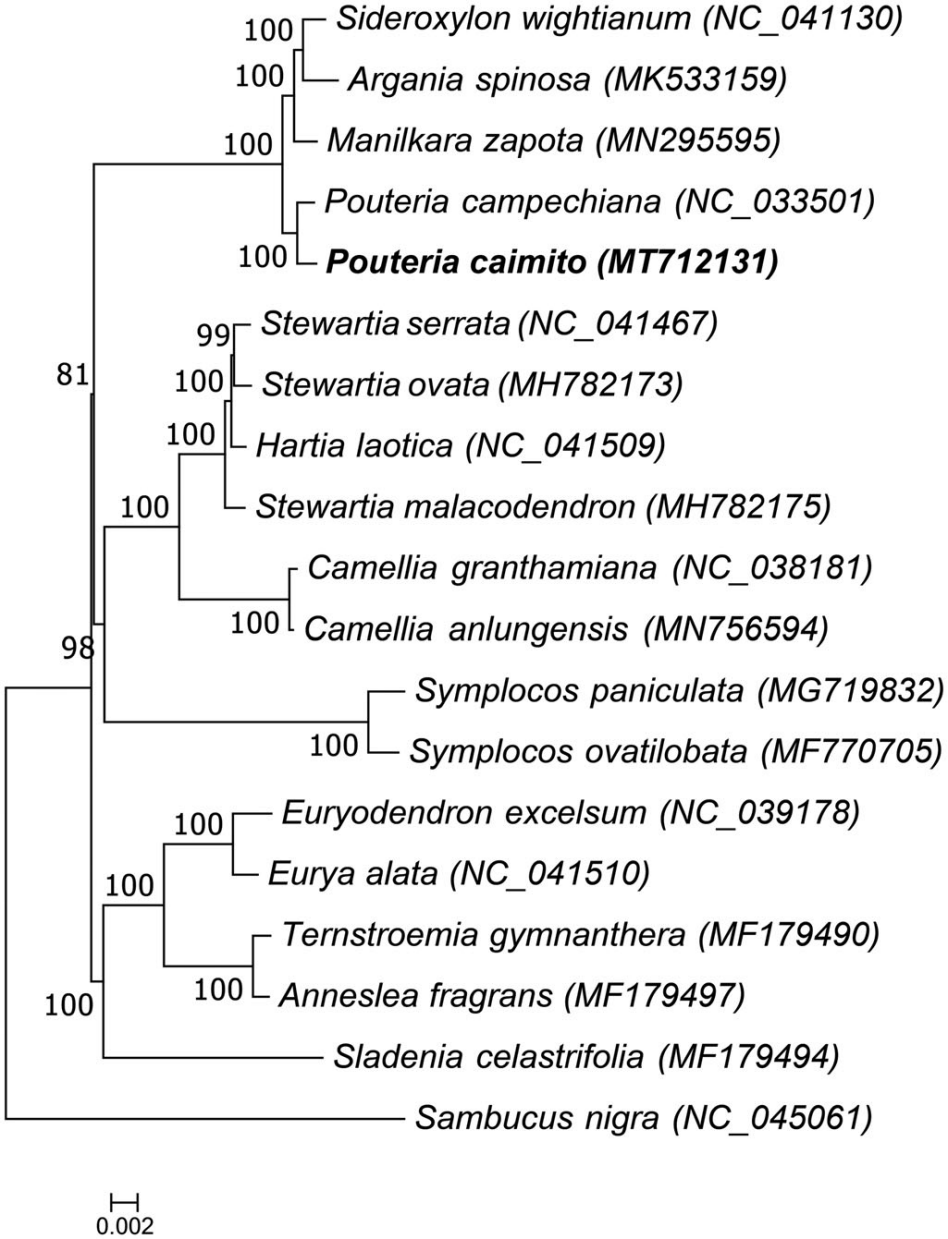
Phylogenetic tree of *Pouteria caimito* and 17 other species of the Ericales order, and *Sambucus nigra* which belongs to the Dipsacales order was used as the outgroup, and the bootstrap value was set to 1000. The 19 species for phylogenetic tree construction are: *Sideroxylon wightianum* (NC_041130), *Argania spinosa* (MK533159), *Manilkara zapota* (MN295595), *Pouteria campechiana* (NC_033501), *Pouteria caimito* (MT712131), *Stewartia serrata* (NC_041467), *Stewartia ovata* (MH782173), *Hartia laotica* (NC_041509), *Stewartia malacodendron* (MH782175), *Camellia granthamiana* (NC_038181), *Camellia anlungensis* (MN756594), *Symplocos paniculata* (MG719832), *Symplocos ovatilobata* (MF770705), *Euryodendron excelsum* (NC_039178), *Eurya alata* (NC_041510), *Ternstroemia gymnanthera* (MF179490), *Anneslea fragrans* (MF179497), *Sladenia celastrifolia* (MF179494), and *Sambucus nigra* (NC_045061).

## Data Availability

The data that support the findings of this study are openly available in GenBank at https://www.ncbi.nlm.nih.gov/Genbank/, reference number MT712131. Raw sequencing reads used in this study have been deposited in SRA with the accession PRJNA658811.
